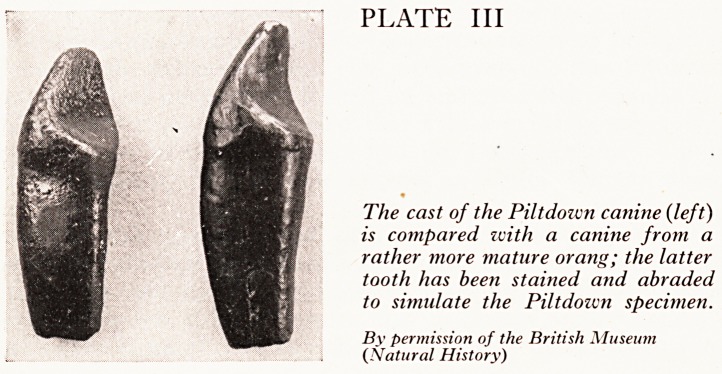# The Elimination of Piltdown Man
*Based on a Presidential Address to the Bristol Branch of the British Dental Association.


**Published:** 1959-01

**Authors:** Hanbury Hazell

**Affiliations:** Dental Surgeon, Southmead Hospital, Bristol


### THE ELIMINATION OF PILTDOWN MAN*

BY

HANBURY HAZELL, L.D.S., R.C.S.

*Dental Surgeon, Southmead Hospital, Bristol*

It is only in recent years that man has become inquisitive about his origin. From early times man was considered unique among all living things on our earth, which in its turn was considered the centre of the universe. With the advance of knowledge, disillusionment followed; for by the discovery that the sun and not the earth was the centre of the universe, Copernicus dealt the first blow to man's egocentricity. Who knows but that visits by spaceships to other planets may inflict a greater and even a more resounding shock?

In the middle of the eighteenth century, Linnaeus divided living things—plants and animals—into groups according to their characteristics and gave each two Latin names so that people of different languages had a common tongue in the field of this particular research. In addition to inventing the binary nomenclature Linnaeus claimed that man did not live in splendid isolation, but was a member of the Primate family! Here was "the unkindest cut of all" to the self-esteem of man, but Linnaeus lessened the insult by classifying man as Homo sapiens thus displaying an unconscious humour or an incurable optimism.

Exactly 100 years ago, the mutability of species was presented by Charles Darwin in his famous book "The Origin of Species through Natural Selection". About the same time, remains of the Neanderthal Man were discovered in a cave near Dusseldorf. These remains were thought to be of an ancestor of modern man. This view however was disbelieved and indeed strongly opposed by German Scientists including Virchow. Subsequently an extremely massive mandible of the Heidelberg man was discovered in Europe and, later in 1894, Pithecanthropus erectus was proclaimed and described by Eugene Dubois, a physician practising in the Dutch East Indies. There is no doubt that Dubois was greatly influenced by Darwin, and his own interest in anthropology led him to his discovery of this Java man. There was no more agreement among the Scientists regarding the importance of Pithecanthropus than there had been about the Neanderthaler and Virchow again led the dissenters.

So we find that at the beginning of the twentieth century extremely little material had been discovered that had any relevance to our enquiries and what there was, was so securely and jealously guarded even from experienced and international authorities, that an expert examination of the original fragments was almost impossible. However a start had been made in beginning to answer the self-imposed question—"Whence came man?"

From the writings of Thomas Huxley, a champion of Darwinism, we find that he considered that the difference in anatomy between man and the Anthropoids was less than that which separated the Anthropoids from the other apes. This, in the nineteenth century, caused an uproar especially among the clergy and the ecclesiastical Primate of all England himself led a furious attack in disputatious argument with Huxley. At this time it was still impossible to estimate the age of rock formation and so the calculation of age of the various fossil discoveries differed according to the personal calculation of each scientist.

There is no doubt that Darwin and Huxley stimulated the interest of all palaeontologists, geologists and anthropologists both professional and amateur. One of these was a Mr. Charles Dawson, a practicing solicitor who was also a very keen,

* Based on a Presidential Address to the Bristol Branch of the British Dental Association.

observant and not un-trained or un-skilled amateur geologist and anthropologist. He practiced at Uckfield in Sussex from 1890 and he became so successful that he obtained appointments as Clerk to the Magistrates, Clerk to the local Council and Steward of several Manors including that of Barkham Manor. This latter appointment occurred in 1898 and it is probable that it was at that time he first visited Barkham Manor at Piltdown near Fletching on his professional business. He was now 33 years of age and had achieved a mature success in his legal work, although his main interest seems to have been in archaeology. His success as an amateur can be deduced from the fact that he already was a Fellow of the Geological Society, Fellow of the Society of Antiquaries and he became a collector for the British Museum after he had presented his well known and documented collection of fossils. In his researches he discovered a source of natural gas which was used at Heathfield for illuminating public premises for many years. At other times he was occupied excavating at Hastings and the Lavant caves, unearthing fossils, identifying metal bearing strata, writing scientific communications and reading papers to various learned Societies. This avid and persistent search engaged Charles Dawson for over thirty years and yet his professional life was as busy as any man's and his law practice was never neglected. In fact it thrived so much that in 1905 he engaged a partner and thereafter was enabled to devote more time to his real love archaeology.

It must be remembered that at the beginning of the twentieth century communication was slow and travelling difficult and tedious so very careful planning was needed before any exploration could be attempted. So it is not surprising that Dawson's interest in the Piltdown gravel pit spread over a number of years. He records that he observed some flints among the gravel which was being used to repair a road near Piltdown Common and learned that they were excavated by workmen from a gravel-bed nearby. He examined the pit but found nothing of interest in it on that occasion but on a further visit, at some unspecified date, a workman gave him a small part of a thick human skull bone and in return no doubt received a suitable and to him more satisfactory token. Many years later (in 1911) in the same gravel pit he discovered another fragment, and the following summer several more, and in all nine skull fragments. A discovery of the greatest importance was apparent to Dawson and he immediately communicated the news to Dr. Arthur Smith Woodward of the British Museum, who examined the collection of fossils, human and animal, and reconstructed a skull from the human remains (Plate I). It was arranged between the two of them to keep the discovery secret until they could announce it to a scientific gathering of the Geological Society in London.

So in December 1912 at such a meeting Dawson and Smith Woodward announced to a large audience the discovery of The Dawn Man. It has been observed how few prehistoric human remains there were that had much significance to the problem "Whence came man?" Here, however were exhibited sufficient fragments to reconstruct a skull together with the lower jaw. Dawson explained how he had found the fossils together with palaeoliths and eoliths in the gravel bed at Piltdown and recalled in detail the various strata from which these fossils derived. From the strata it was possible to estimate approximately the age of our primitive ancestor. It also was possible of course that some fossils may have been washed down into the different layers of gravel of which there were three, but it was thought that they represented the Early Ice Age or possibly earlier. Attention was then drawn to the man-made palaeoliths to show that this Piltdown man could make tools because both were buried together.

It was then left to Smith Woodward to present to the impatient and excited audience the animal and human fragments; to describe each in detail and to give his reason[illegible] for their identification. The animal fragments, chiefly teeth, he recognized as belongin[illegible] to horse, deer and hippopotamus. The reconstruction of the skull from the nin[illegible] pieces of cranial bone and a portion of mandible with two molar teeth *in situ*, wa[illegible] explained and the complete replica was exhibited. The skull was large, with a hig[illegible]

forehead and a cranial capacity of about 1,350 c.cm. The bony wall was very thick—about 10 m.m. As we know that the modern European has a cranial capacity very little larger, it can be appreciated how easily Piltdown man was accepted most readily as regards this characteristic feature but the mandible presented many objections. The fragment consisted of the side of a lower jaw extending from just in front of the first molar backwards but with the condyle missing. The first and second molar teeth were firmly in their sockets and the occlusal surfaces worn flat, which presupposes that the mandible must have been freely movable in more than one plane as in modern man. The lower border of the jaw went further forward although the chin itself was missing as was the canine tooth which would have been invaluable. As presented complete, this Dawn Man had a human cranium but an ape-like jaw. This combination coincided with Darwin's prediction and what the anthropologists were convinced would be discovered.

Professor Waterston refused to his dying day to believe that the two came from the same creature and suggested that the jaw was that of a chimpanzee. There were the flat molars however and no monkeys or apes had worn molars such as these. There was almost universal agreement about the Piltdown Man and Dawson was honoured when it was named Eoanthropus Dawsoni. There was just one thing that Professor Arthur Keith, who at that time was Conservator at the Royal College of Surgeons, did not accept and that was the interpretation of the canine tooth. Professor Elliott Smith, an eminent authority on the brain, after careful examination of the brain cast declared it human and slightly larger than the reconstruction. Keith differed from both Elliott Smith and Smith Woodward in their reconstruction of the cranium and spent valuable time in argument.

In 1913 Father Teilhard a French Priest working with Dawson found a lower canine tooth in the same gravel bed at Piltdown and it corresponded almost exactly with the reconstructed one Smith Woodward had designed previously. Dr. A. S. Underwood took X-rays of the canine and the picture showed that great wear had taken place on the crown, so much so that the pulp had laid down secondary dentine and receded. One curious feature was the pulp canal wide open at the apex of the root. Mr. C. Lyne, a dental surgeon, in a paper he read at the Royal Society of Medicine pointed out that to bear the mark of such attrition the animal must be mature but the tooth was young as witness the wide open root apex. Underwood who was a well known dental anatomist forcefully and scornfully opposed this view and his interpretation was accepted by Keith unfortunately as well as by other experts.

If this were not enough, further evidence of antiquity was forthcoming as in 1914 a fossil bony club was found by Dawson at the original site and the following year 1915 he unearthed a molar tooth and part of a cranial bone at Sheffield Park a few miles from Piltdown. This was accepted as the second Piltdown Man. Organized digging took place in 1916 but Dawson took no part as he was suffering a disease to which he succumbed later that year. Nothing further was discovered but already there was a sufficiency to establish this primitive man as Darwin's "missing link". Not that there was unanimous agreement even at that time—far from it. Furious argument and dissension raged among the scientists. Some subscribed to the monistic view that the cranium and the mandible belonged to the same creature; others to the dualistic belief of the human cranium and the ape's jaw, while a few were irreconcilable. The British public took no interest in the matter for it was by then engaged in World War I.

After the war was over, search was made in various parts of the world for evidence of prehistoric man. Energy and perspicuity were rewarded when the second Java Man was discovered, the Solo Man and in China, the Pekin Man. In South Africa too, many specimens were discovered notably the Rhodesian Man. The striking thing about all these later discoveries was that they possessed simian characteristics in every way except the jaw and teeth, which were similar to those of modern man. This appearance would be the complete reversal of the human-like cranium and ape-like jaw of Piltdown. Piltdown Man was alone, the odd man out. The parallel theory was now evoked to suggest that two distinct forms developed simultaneously and there was confusion as to which was the ancestor of Homo sapiens.

In 1948 Dr. Kenneth Oakley of the British Museum described a method by which the age of a bone could be ascertained according to the amount of fluorine it contained. The amount of fluorine absorbed into the chemical structure depended upon the time the bone remained buried in the earth. In subsequent years he applied the fluorine test to the Piltdown bones and estimated that they were not nearly so old as had been believed, and immediately refuting what Sir Arthur Keith had written— "Piltdown Man represents the earliest specimen of humanity yet discovered". It is worth while recording that Mr. Alvan Marston, a Dental Surgeon, had long maintained that the jaw was simian and the mandibular right canine tooth was in fact a left maxillary canine belonging to an Orang-utan!!

Thus Eoanthropus seemed to have become an impossibility or so thought Dr. J. S. Weiner, an anthropologist at Oxford University. If the jaw had been treated to make it appear older than it was, perhaps the teeth had received treatment too and recently at that. He imparted his thoughts to Professor le Gros Clark who supported him in his effort to solve the apparent paradox. They re-examined the casts and with Dr. Oakley were permitted to examine the originals at the British Museum. Artificial scratches were observed on the occlusal surfaces of the molar and also on the canine and suspicion of fraud became so great that it was not difficult to obtain permission to subject Piltdown Man to a bombardment of comprehensive scientific tests. Dr. Oakley sought the aid of his colleagues, all expert in their own particular fields of research, and detection began.

The fluorine test showed that the skull bones were considerably older than the mandible so that the cranium and jaw belonged to two different beings. With increasing age while the fluorine in fossil bone increases, the nitrogen content decreases. With this test however the jaw and teeth contained a high nitrogen content and the cranial fragments a little. (It should be mentioned that some authorities do not accept this as conclusive evidence because Dr. A. J. Clement has stated that the organic matter in Homo rhodesiensis is unchanged.) The possibility of forging a portion of mandible with two molar teeth *in situ* became a probability when the right side of an orang-utan mandible was broken to the same dimensions and the occlusal surfaces of the molars ground flat as exhibited in the Piltdown specimen (Plate II). A canine tooth of an orang-utan was similarly abraded to simulate the attrition shown in the Piltdown tooth (Plate III). Suitably stained the comparative specimens evinced a striking resemblance. The brown staining of the specimens proved to be artificial especially in the canine where it was very superficial. The teeth had been artificially abraded to simulate natural wear and the canine so excessively as to expose the pulp chamber, the exposure being plugged by a plastic material. This was demonstrated clearly by X-ray pictures as was the wide open root apex and the loose sandy particles in the pulp canal. The occlusal surfaces of the molars were not in the same plane as one would expect. Samples of powdered bone were examined by X-ray crystallography and even the electron microscopes and the Geiger counter were brought into action.

The results of all the exhaustive tests convinced everyone that Piltdown Man was ill-gotten, and had been deliberately foisted on and accepted by most of the expert scientists. The bitter truth was revealed that Piltdown Man was a forgery. The extraordinary thickness of the skull bone had not been explained but it has been suggested as pathological. In any case negroes in Dutch Guiana possess equally thick skulls.

Who perpetrated this gigantic fraud with such care and patience? It must have been someone with sufficient specialised knowledge. He must have had the capacity and the opportunity and certainly possessed a strong enough motive. From all accounts Dawson was not at all a popular man in Sussex archaeological circles and no doubt had many enemies, possibly through jealousy of his discoveries. One of

PLATE I

*By permission of the British Museum (Natural History)*

*The Dawn Man of Sussex*—Eoanthropus dawsoni.
(*A composite reconstruction of brain-case and jaw.*)

[*Face page 4*

### PLATE II

*By permission of the British Museum (Natural History)*

*The inner aspect of the Piltdown mandible (lower) is shown for comparison with a mandible from a female orang-utan (upper); the upper specimen has been broken and the teeth abraded to simulate the Piltdown specimen. (Prepared by L. E. Parsons)*

### PLATE III

*The cast of the Piltdown canine (left) is compared with a canine from a rather more mature orang; the latter tooth has been stained and abraded to simulate the Piltdown specimen.*

*By permission of the British Museum (Natural History)*

these may have sought revenge for some considered slight by planting the forgeries in the gravel bed where Dawson would find them. To be able to do so on so many occasions and over a period of years without discovery he would indeed be lucky. However improbable this may be, it is a possible explanation and maybe a charitable one. Dawson is known to have stained certain fragments with dichromate to harden them, as he explained. If he were not the culprit he certainly assisted in the forgery by this action. Smith Woodward and Teilhard are completely above the slightest suspicion as they had no part in and even were not available during most of the discoveries. They were men of the highest integrity. This circumstantial evidence is not conclusive proof that Dawson was the guilty person, but clearly suspicion invests him with a large measure of responsibility. It may well be that the most culpable were the eminent scientists who so eagerly accepted Piltdown Man as an answer to their preconceived ideas without the critical and stringent analyses of which they were capable.

In the headquarters of the Geological Society in London there was a painting depicting those most associated with the discovery and identification of Piltdown Man. It was painted in 1915 and shows Professor Arthur Keith examining the skull while Professor Elliott Smith is drawing attention to a certain detail. Half a dozen others are grouped around with Dawson and Smith Woodward prominent and in the distance a portrait of Charles Darwin hangs on the wall. Maybe this painting does not occupy such an honoured position today.

The energy expanded over forty years by scientists on Piltdown Man might have been employed more profitably in the pursuit of their knowledge of legitimate ancestors. Although we are a long way from understanding how man came to his present state, anthropologists and palaeontologists must be greatly relieved that Piltdown Man has been eliminated from their researches and this enigma, as Keith called him, banished from serious consideration.

### ACKNOWLEDGMENTS

I wish to thank Professor J. S. Weiner for his ready kindness in allowing me to reproduce the illustrations of the composite reconstruction of brain-case and jaw of Piltdown Man and the canine teeth compared, as published by Oxford University Press, London, in his book "The Piltdown Forgery". I also am grateful that the illustrations of the Piltdown and Orang-utan mandible are included: "By permission of the British Museum (Natural History)", and by courtesy of the Trustees.

### REFERENCES

Clement, A. J. (1958). *Brit. dent. J.*
Keith, Sir Arthur. (1925). *The Antiquity of Man.* Williams and Norgate, London.
Lyne, C. W. (1916). Significance of the Radiographs of Piltdown Man. *Proc. Roy. Soc. Med.*
Underwood, A. S. (1913). "The Piltdown Skull" *Brit. J. Dent. Sci.*
Von Koenigswald, G. H. R. (1956). *Meeting Prehistoric Man.* Thames and Hudson, London.
Oakley, K. P. (1948). Fluorine and the Relative Dating of Bones. Advancement of Science, **16.**
Weiner, J. S. (1955). *The Piltdown Forgery.* Oxford University Press, London.
Weiner, J. S., Oakley, K. P. and Le Gros Clark, W. E. (1953). "The solution of the Piltdown Problem" *Bull. Brit. Mus. (Nat. Hist.) Geol.*

## Figures and Tables

**Figure f1:**
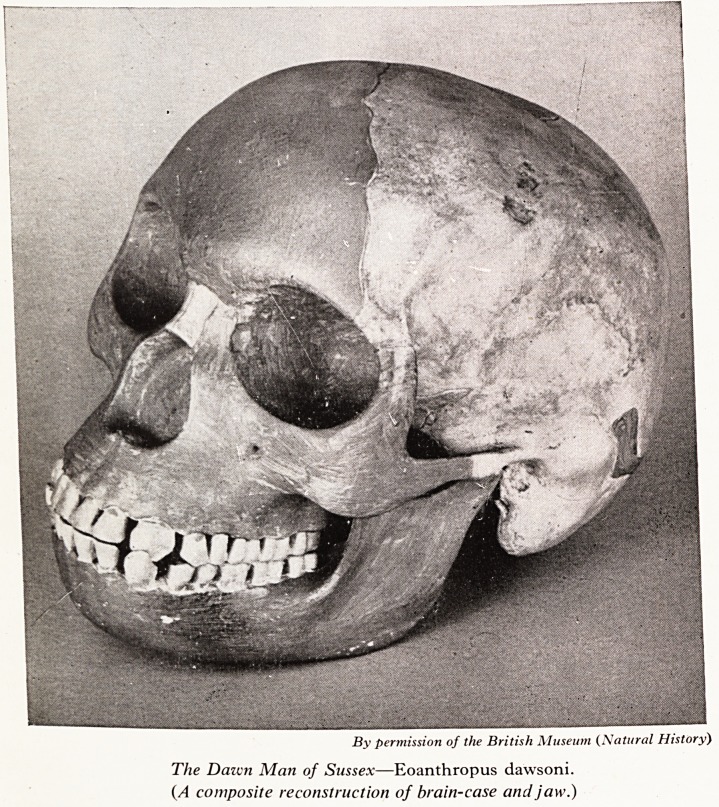


**Figure f2:**
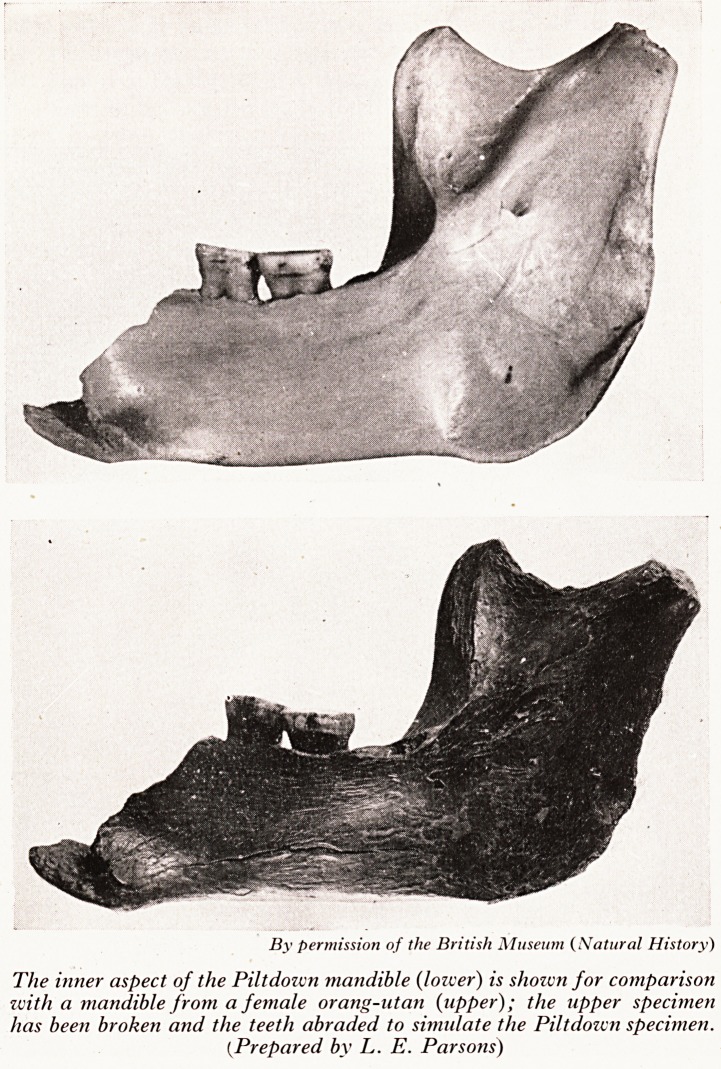


**PLATE III f3:**